# Fractionation of Aspen Wood to Produce Microcrystalline, Microfibrillated and Nanofibrillated Celluloses, Xylan and Ethanollignin

**DOI:** 10.3390/polym15122671

**Published:** 2023-06-13

**Authors:** Boris N. Kuznetsov, Anna I. Chudina, Aleksandr S. Kazachenko, Olga Yu. Fetisova, Valentina S. Borovkova, Sergei A. Vorobyev, Anton A. Karacharov, Elena V. Gnidan, Elena V. Mazurova, Andrey M. Skripnikov, Oxana P. Taran

**Affiliations:** 1 Institute of Chemistry and Chemical Technology, Krasnoyarsk Scientific Center, Siberian Branch, Russian Academy of Sciences, Akademgorodok 50/24, Krasnoyarsk 660036, Russiafou1978@mail.ru (O.Y.F.); bing0015@mail.ru (V.S.B.); karacharov@icct.ru (A.A.K.); egnidan@gmail.com (E.V.G.); len.mazurowa@yandex.ru (E.V.M.); and-skripnikov@yandex.ru (A.M.S.); taran.op@icct.krasn.ru (O.P.T.); 2 Department of Non-Ferrous Metals and Materials Science, Siberian Federal University, pr. Svobodny 79, Krasnoyarsk 660041, Russia

**Keywords:** aspen wood, fractionation, xylan, ethanollignin, microcrystalline, microfibrillated and nanofibrillated celluloses

## Abstract

A new method for extractive-catalytic fractionation of aspen wood to produce microcrystalline (MCC), microfibrillated (MFC), nanofibrilllated (NFC) celluloses, xylan, and ethanollignin is suggested in order to utilize all of the main components of wood biomass. Xylan is obtained with a yield of 10.2 wt.% via aqueous alkali extraction at room temperature. Ethanollignin was obtained with a yield of 11.2 wt.% via extraction with 60% ethanol from the xylan-free wood at 190 °C. The lignocellulose residue formed after the extraction of xylan and ethanollignin was subjected to catalytic peroxide delignification in the acetic acid-water medium at 100 °C in order to obtain microcrystalline cellulose. MCC is hydrolyzed with 56% sulfuric acid and treated with ultrasound to produce microfibrillated cellulose and nanofibrillated cellulose. The yields of MFC and NFC were 14.4 and 19.0 wt.%, respectively. The average hydrodynamic diameter of NFC particles was 36.6 nm, the crystallinity index was 0.86, and the average zeta-potential was 41.5 mV. The composition and structure of xylan, ethanollignin, cellulose product, MCC, MFC, and NFC obtained from aspen wood were characterized using elemental and chemical analysis, Fourier-transform infrared spectroscopy (FTIR), X-ray diffraction (XRD) analyses, Gas chromatography (GC), Gel permeation-chromatography (GPC), Scanning electron microscopy (SEM), Atomic force microscopy (AFM), Dynamic light scattering (DLS), Thermal gravimetric analysis (TGA).

## 1. Introduction

Wood biomass is mainly composed of cellulose, hemicelluloses and lignin; this is a renewable feedstock for the production of biofuels, valuable chemicals and composites [1,2,3,4]. Promising technologies for the processing of the wood to in-demand products are based on the fractionation of the main components of woody biomass [5,6]. Catalytic peroxide fractionation of wood biomass was effectively used for the manufacturing of microcrystalline cellulose (MCC), hemicelluloses, organosolvent lignin-based enterosorbents, as well as aromatic acids and monosaccharides [7,8]. Catalytic reductive fractionation allows monomer phenols to be obtained from lignin while the major part of the cellulose is preserved [9,10].

Organosolvent methods that are used for cellulose production generate organosolv lignin, which is environmentally less dangerous than traditional technical lignins [11,12]. Organosolv lignins, in comparison to technical lignins, are characterized by a lower molecular mass and a rather narrow molecular mass distribution. They are readily soluble in organic solvents, structurally close to native lignins, and free of inorganic components (sulfur, nitrogen, etc.). All of this facilitates their further catalytic processing [13,14]. Organosolv lignins have the potential to be used as natural antioxidants in the cosmetics and food industries [15], as a component in UV-blocking sunscreens [16], and as enterosorbents [8]. Hardwood contains a valuable polysaccharide xylan (up to 19 wt.%) which is used for the synthesis of xylose, xylite, other polyatomic alcohols, organic acids, nutrient yeast, and a number of other products [17]. Xylan is used in the food, cosmetics, and pharmaceutical industries [18,19,20], and it has good prospects for application in targeted drug delivery systems [21,22]. New highly hydrophobic coatings [23] and multifunctional bioplastics [24] based on xylan are being developed. Owing to their biocompatibility and biodegradable nature, nanocelluloses from lignocellulose biomass are increasingly used for the manufacturing of new nanocomposite materials [25,26]. Low density, high mechanical properties, a larger surface area, and other useful properties are characteristic of the nanocellulose materials [27]. In recent years, greater attention has been given to nanocellulose applications such as paper, packaging materials including film packs [28,29], and insulating materials [30].

Among nanocellulose materials, there are microfibrillated celluloses (MFC) where the fibrils are 10–100 nm in thickness and 0.5–50 µm in length; there are also nanofibrillated celluloses (NFC) that are 5–30 nm thick and longer than 250–1500 nm fibrils, and nanocrystalline celluloses with 3–10 nm thick and longer than 15–50 nm fibrils [31].

Nanocelluloses are obtained from various types of lignocellulose raw materials. The yield and structure of nanocelluloses depend on the nature of lignocellulose used (softwood or hardwood, for example). Moreover, the chemical composition of woody biomass changes not only with variations in the type of tree, but also along and across the trunk of a tree [32].

Aspen is among the main forest-forming species in Russia; it is a valuable renewable resource for the production of in-demand chemicals. The methods for production of nanofibrillated celluloses from aspen wood are described in the literature: the wood is pretreated under severe conditions, for example TEMPO-oxidation [33], or steam treatment at high pressures and temperatures [34]. When these methods are used, the wood lignin and hemicelluloses are being lost. It has been suggested to integrate the processes of wood autohydrolysis followed by the delignification of the autohydrolysed material in formic acid; however, at these conditions, the hemicellulose fraction is lost and the cellulose quality deteriorates [35].

The fractionation of a wood biomass into cellulose and liquid products enriched with methoxyphenols (mainly propylsyringol and propylguaiacol) was performed using the wood hydrogenation with hydrogen in ethanol at 250 °C in the presence of Ru/C and Pt/ZrO_2_ catalysts [36].

In the present work, we suggest a new method for the fractionation of aspen wood to xylan, ethanollignin, microcrystalline, and microfibrillated and nanofibrillated celluloses. The method is based on the integration of extractive and catalytic processes. To characterize the obtained products, we used elemental and chemical analysis, Fourier-transform infrared spectroscopy (FTIR), X-ray diffraction (XRD) analyses, Gas chromatography (GC), Gel permeation-chromatography (GPC), Scanning electron microscopy (SEM), Atomic force microscopy (AFM), Dynamic light scattering (DLS), and Thermal gravimetric analysis (TGA).

## 2. Materials

We used the sawdust (fraction 1.0–2.0 mm) of aspen wood (*Populus tremula* L.), harvested in the vicinity of the city of Krasnoyarsk as the initial raw material. The sawdust was pre-dried in a drying oven at 60 °C for 16 h. The following reagents were purchased from Khimreaktivsnab (Ufa, Russia) for the work: glacial acetic acid (97%), ethanol (96%), sulfuric acid (95–97%), sodium hydroxide (98%), and hydrogen peroxide (35%).

### 2.1. Preparation of Chemicals from Aspen Wood

Xylan was separated by treating aspen sawdust with the aqueous 4% NaOH solution by the method described elsewhere [37]. Resinous substances were pre-removed by boiling the sawdust with aqueous 50% ethanol (hydromodulus 40) for an hour. The sawdust was then extracted with a 4% sodium hydroxide solution (hydromodulus 40) at room temperature under stirring for 6 h. The solution was separated by filtering and neutralized with acetic acid. Xylan was precipitated by adding double the volume of ethanol, after which the solution was kept at +4 °C for 16 h, decanted, and then double the volume of ethanol was added. The precipitated xylan was isolated by centrifuging (8000 rpm, OHAUS Frontier 5816), frozen at −18 °C, and lyophilized in an Iney-6 drier. The lignocellulose residue was dried at 60 °C for 16 h.

The ethanollignin was extracted with 60% ethanol from the xylan-free aspen wood at 190 °C for 3 h using a ChemRe SYStem R-201 (ChemRe SYStem Inc., Anyang, Republic of Korea) autoclave-type reactor by the method described elsewhere [38]. The dissolved ethanollignin was precipitated with cold water (+4 °C), filtered from the solution, and dried to a constant weight at 50 °C. The resulting cellulose product (CP) was dried at 60 °C for 16 h.

Microcrystalline cellulose (MCC) was obtained via peroxide delignification of the cellulose product (CP) in the ‘acetic acid—hydrogen peroxide—water’ medium over 4 h; the process was carried out at 100 °C and with hydromodulus 15 in the presence of a H_2_SO_4_ catalyst (1% of the wood weight) by the method described elsewhere [7]. The produced MCC was filtered from the solution, washed with distilled water to reach the neutral reaction of the washing water, dried at 60 °C, and sieved (with a mesh size of 0.5 mm).

In order to obtain microfibrillated and nanofibrillated celluloses, a MCC sample (0.5 g) was treated with a 56% H_2_SO_4_ solution (12 mL) at 45 °C for an hour under continuous stirring. The hydrolysis reaction was inhibited by adding 120 mL of cold distilled water. The resulting mixture was allowed to settle for 12 h and centrifuged (12,000 rpm, OHAUS Frontier 5816). The solution was decanted. Another portion of water was added to the precipitate and mixed; the mixture was centrifuged and decanted again. To prevent overheating, 20 mL of distilled water and distilled water ice was added to the precipitate. The mixture was exposed to an ultrasonic treatment for 9 min using a UZTA-1/22-OPD Volna-M apparatus (up to 1000 W power, with an ultrasonic frequency of 22 kHz). The centrifugate (NFC) and the precipitate (MFC) were frozen at −18 °C and lyophilized in an Iney-6 drier.

### 2.2. Product Characterization

The contents of the lignin in the wood, the cellulose product, and the microcrystalline cellulose were determined using hydrolysis with sulfuric acid (72 wt.%) at 20 °C for 2.5 h, followed by the dilution of the reaction mixture with water and boiling for an hour [39]. The contents of cellulose in the wood and in cellulose products were determined by the Kürschner method [40]. In order to determine the contents of readily hydrolyzed polysaccharides, samples were hydrolyzed with 2% HCl at 100 °C for 3 h [41]. The gas chromatographic identification of monosaccharides in the hydrolysate was performed using a VARIAN-450 GC gas chromatograph with a flame-ionizing detector and a VF-624ms capillary column (30 m length, 0.32 mm inner diameter). A hydrolysate sample was pre-derivatized to form trimethylsilyl derivatives [42]. The contents of the extractants in the wood were determined by extraction with an alcohol-toluene mixture (1:2 *vol*/*vol*) for 8 h using a Soxhlet apparatus. The ash content was determined using the standard TAPPI T211 method based on calcining the sample in air at 525 °C.

The degree of polymerization (DP) of the cellulose products was determined by measuring the characteristic viscosity in an iron sodium tartaric complex (ISTC) at 20 °C using a VPZh-2 type capillary viscometer [41].

In order to identify the chemical composition of xylan, it was hydrolyzed with 10% sulfuric acid at 105 °C for 2.5 h [43]. Gas chromatography was used for determining the monosaccharide content in the xylan hydrolyzate.

The FTIR spectra of the samples was acquired at 4000–400 cm^−1^ using a Tensor 27 IR-Fourier spectrometer (Bruker, Ettlingen, Germany). The samples to be studied were prepared in the form of pressed pellets containing 4 mg of the sample in the potassium bromide matrix.

Gel permeation-chromatography via an Agilent 1260 Infinity II Multi-Detector GPC/SEC Systemchromatograph and refractive index detection was conducted to determine the weight-average molecular mass (M_w_) and the number-average molecular mass (M_n_) polydispersity (PD) of the ethanollignin of aspen wood. A PLgel Mixed-E column with tetrahydrofuran stabilized with 250 ppm butylhydroxytoluene as the eluent was used for separation. The columns were calibrated using polystyrene (PS) polydisperse standards (Agilent, Santa Clara, CA, USA). The eluent was fed at the rate of 1 mL/min, with a 100 mcl sample being injected. The Agilent GPC/SEC MDS program package was used for the data collection and processing.

XRD patterns of the cellulose samples were acquired using a PANalytical X’Pert Pro diffractometer with CuKα (λ = 0.154 nm) radiation at the 2θ range between 10 and 50° in 0.01° intervals in a cell of 2.5 cm in diameter. The crystallinity index (*CI*) was calculated from the ratio of the height between the intensity of the crystalline peak (*I*_002_ − *I_AM_*) to the total intensity (*I*_002_) after the subtraction of the background signal:

(1) $$CI=\frac{I_{002}-I_{AM}}{I_{002}},$$

where *I*_002_ is the height of peak 002; and *I_AM_* is the height of the minimum between peaks 002 and 101 [44].

Surface morphology of cellulose samples was studied using a TM-4000 scanning electron microscope (Hitachi, Tokyo, Japan) equipped with an energy-dispersive analyzer SwiftED3000 (Oxford Instruments Inc., Bognor Regis, UK) with the accelerating voltage of 15 kV and a resolution of 20 µm.

The hydrodynamic diameter of NFC particles was measured by dynamic light scattering using a Zetasizer Nano ZS spectrometer (Malvern Instruments Ltd., Malvern, UK). The prepared suspension contained 0.1 mg/mL of cellulose. Measurements of zeta-potentials of weighed NFC particles were based on the electrophoretic mobility in polycarbonate cells with Pd electrodes at 20 °C without the addition of a background electrolyte or pH correction.

The structure of the NFC film was characterized by atomic force microscopy in semi-contact mode using a multimode scanning probe Solver P 47 (NT-MDT, Moscow, Russia) microscope equipped with a 14 mcm scanner. The application of a drop of NFC suspension (0.1 mg/mL) on the surface of highly-oriented pyrolytic graphite was followed by drying and rinsing with bidistilled water. The scanning rate was 40–55 mcm/s, with a 256 × 256 point being scanned on the scanned surface.

A Vario El Cube ELEMENTAR (Langenselbold, Germany) analyzer was used for establishing the elemental compositions of MCC, MFC, and NFC samples during controlled sample burning in pure oxygen at 1200 °C.

A thermal analysis of the MCC, MFC, and NFC samples was conducted using an STA 449 F1 Jupiter instrument (NETZSCH, Bavaria, Germany); the samples were heated in argon from 30 to 800 °C at the rate of 10 °C/min. The data obtained were processed using the NETZSCH. ProteusThermalAnalysis.5.1.0 program package.

The kinetics of the thermal decomposition of cellulose samples was studied by the model-fitting Coats-Redfern method by assuming the first order reaction [45,46].

The main kinetic parameter, activation energy, was calculated based on a generalized fundamental expression (2) for the rate of a solid-phase reaction under non-isothermal conditions:

(2) $$\frac{d\alpha }{dT}=\frac{A}{\beta }e^{\frac{-E}{RT}}f\left(\alpha \right),$$

where *A* is the pre-exponential factor (s^−1^), *β* is the heating rate (degree·min^−1^), *E* is the activation energy (J·mol^−1^), *R* is the universal gas constant (J·mol^−1^·K^−1^), *T* is the temperature (K), and *f*(*α*) is the mathematical model of a dimensionless kinetic function depending the reaction type and mechanism. The conversion of matter $\alpha =\frac{m_{i}-m}{m_{i}-m_{f}}$, where *m_i_* and *m_f_* are the initial and final matter mass, and *m* is the matter mass at the measurement point.

The main Coats-Redfern equation is as follows:

(3) $$ln\left[\frac{g(\alpha )}{T^{2}}\right]=ln\left(\frac{AR}{\beta E}\right)-\frac{E}{RT},$$

where *g*(*α*) is the integral function *f*(*α*) (see Equation (2)).

The graphical interpretation of Equation (3) for the kinetic parameters of thermogravimetric curves of the first order reaction is based on the following equation:

(4) $$ln\left[-\frac{ln(1-\alpha )}{T^{2}}\right]=ln\left(\frac{AR}{\beta E}\right)-\frac{E}{RT}$$

The dependence of $ln\left[-\frac{ln(1-\alpha )}{T^{2}}\right]$ on 1/*T* is approximated with a linear function. The activation energy is determined based on the slope of the resulting lines.

## 3. Results and Discussions

### 3.1. Extractive Catalytic Fractionation of Aspen Wood

**Figure 1:**
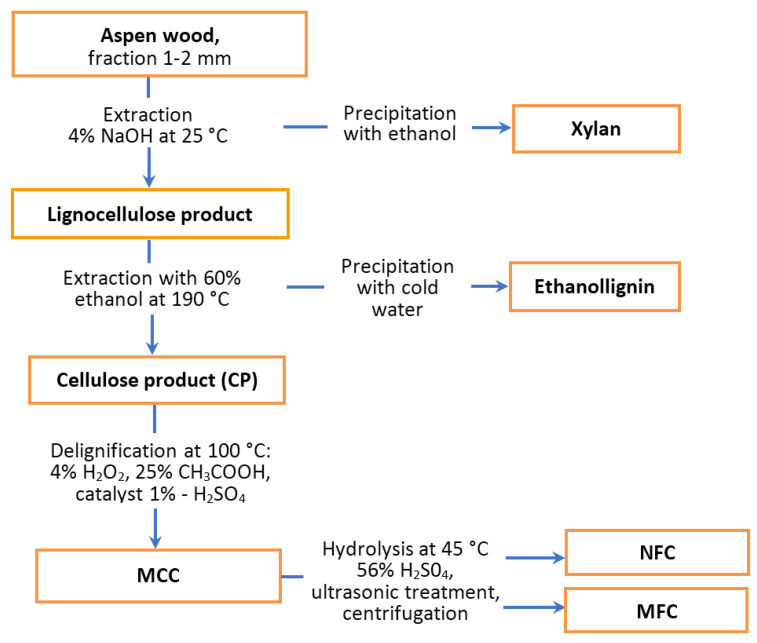
Schematic of aspen wood fractionation.

The aspen wood used in the work had the following chemical composition: 44.4 wt.% cellulose, 23.0 wt.% lignin, 19.2 wt.% hemicelluloses, 8.5 wt.% extractants, and 0.4 wt.% ash. The suggested procedure for fractionation of aspen wood to valuable chemicals includes stages of isolation of xylan using an alkali treatment of the wood, the isolation of ethanollignin by treating the xylan-free wood with ethanol, the separation of microcrystalline cellulose (MCC) from the wood after extraction via catalytic peroxide delignification, the conversion of MCC to microfibrillated cellulose (MFC), and nanofibrillated cellulose (NFC) using sulfuric acid hydrolysis and ultrasonic treatment and centrifugation for the separation of the MFC and NFC (Figure 1).

The compositions and structures of the products from wood were established using elemental, chemical and physicochemical analyses.

### 3.2. Isolation, Composition, and Structure of Xylan

The basic methods for xylan separation are acid and alkali hydrolysis, as well as the autohydrolysis of lignocellulose materials [47,48,49].

The main disadvantage of autohydrolysis is that the formation of undesirable side products such as furfural and hydroxymethylfurfural makes the process of xylan purification very difficult [49]. The acid pretreatment of the wood causes the destruction of xylan, the formation of toxic compounds, and the corrosion of the equipment [48]. The alkali pretreatment of the lignocellulose is the preferable method for xylan extraction for pharmaceutical and food applications [50]. Alkali reagents taken in moderate concentrations favor the cleavage of the hydrogen bonds of the lignin-cellulose and the hemicelluloses-cellulose, as well as the ester bonds between the lignin and the hemicelluloses to provide the effective solubilization of xylan [51].

The chemical composition of xylan extracted from aspen wood was characterized on the basis of the contents of monosaccharides formed during xylan hydrolysis with 2% HCl at 100 °C. The hydrolyzate comprised 98.7 rel% of xylose, 0.3 rel% of mannose, 0.4 rel% of galactose, 0.5 rel% of glucose, and the arabinose was not identified. Hence, the extracted xylan primarily contains xylose units.

The elemental composition of xylan from aspen wood is close to 99% pure commercial xylan [52], and is almost identical to the elemental composition of xylan obtained by the alkali extraction of birch wood [53]. The data on the elemental composition of xylan extracted from aspen wood are summarized in Table 1.

**Figure 2:**
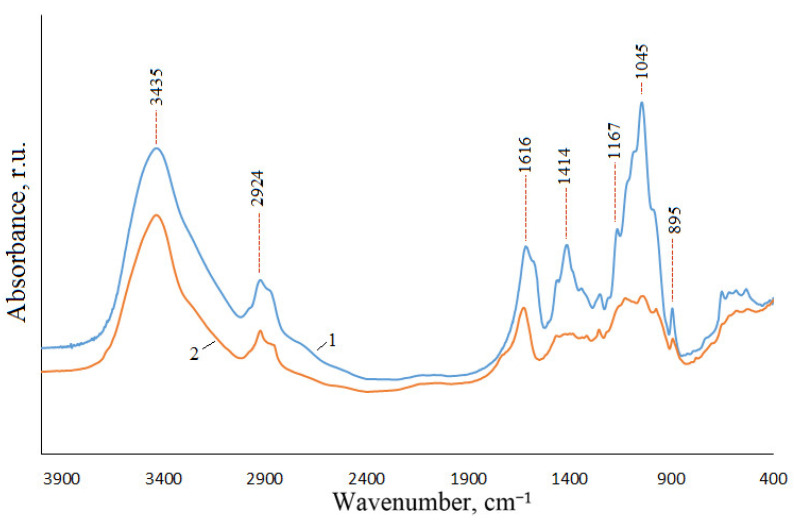
IR spectra of xylan from aspen wood (1) and commercial xylan from beech wood (2).

In general, the infrared spectrum of xylan extracted from aspen wood matches to that of the commercial xylan extracted from beech wood (Figure 2). The absorption band at 3435 cm^−1^ is assigned to stretching vibrations of the O-H bond, at 2924 cm^−1^, to stretching vibrations of the C-H bond, at 1045 cm^−1^, to stretching vibrations of the C-C bond, at 1167 cm^−1^, to stretching vibrations of the C-O-C bond [54]. The absorption band at 895 cm^−1^ relates to vibrations of the β-glycoside bond, at 1616 cm^−1^ and 1414 cm^−1^, to vibrations of bonds in the –COO- group of the sodium salt of glucuronic acid.

Thus, the composition of xylan extracted from aspen wood is close to the composition of xylans from the wood of birch and beech.

### 3.3. Isolation, Composition and Structure of Ethanollignin

The methods of organosolvent delignification with various organic solvents are used for the separation of reactive organosolvent sulfur-free lignin [55,56,57].

Data on the composition and structure of ethanollignin from aspen wood were obtained using IR spectroscopy, elemental analysis, and gel penetrating chromatography. The elemental composition of ethanollignin extracted from alkali treated aspen wood (Table 2) is close to the composition of ethanollignin obtained via high-pressure extraction from poplar dust at 180 °C using aqueous ethanol.

**Figure 3:**
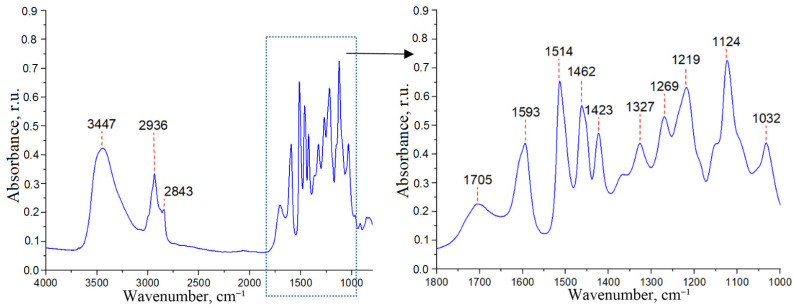
The IR spectrum of ethanollignin from aspen wood.

The IR spectrum of ethanollignin isolated from xylan-free aspen wood (Figure 3) is similar to the spectrum of ethanollignin extracted from unprocessed aspen wood [59].

The band at 3447 cm^−1^ is assigned to the stretching vibrations of OH-groups associated through hydrogen bonds. Bands at 2936 cm^−1^ and 2843 cm^−1^ relate to stretching vibrations of the C-H bonds in the methyl and methylene groups, respectively, and the band at 1462 cm^−1^ is assigned to asymmetric bending vibration in methyl groups. The band at 1705 cm^−1^ is characteristic of stretching vibrations of C=O bonds in ketones, carbonyls, and ester groups; bands at 1593 cm^−1^, 1514 cm^−1^, and 1423 cm^−1^ are characteristic of skeletal vibrations of aromatic rings. Bands at 1327 cm^−1^ and 1219 cm^−1^ are assigned to skeletal vibrations, and the band at 1124 cm^−1^ is assigned to the in-plane bending vibrations of the C-H bonds of the syringyl ring. The absorption bands at 1269 cm^−1^ and 1032 cm^−1^ are characteristic of the skeletal and in-plane vibrations of the guaiacyl ring. The fact that the absorption bands of syringyl rings are more intense than those of guaiacyl rings indicates the predominance of syringyl structures in the ethanollignin of aspen wood.

**Figure 4:**
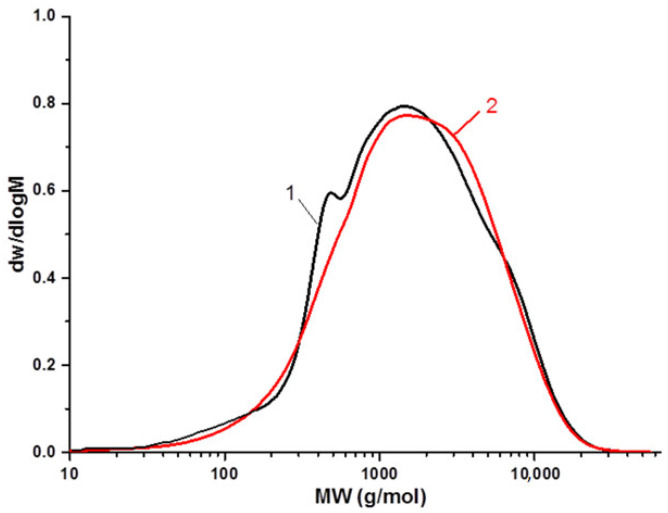
The molecular mass distribution of ethanollignin from xylan-free aspen wood (1) and from initial aspen wood (2).

The molecular masses and molecular mass distributions of the ethanollignin extracted from the initial aspen wood and from the xylan-free wood were studied using a gel-penetrating chromatographic technique. The data obtained show (Table 3) only a minor difference between the weight-average molecular masses (M_w_) of these ethanollignin samples of about 2700 g/mol, which agrees with the data previously obtained from the ethanollignins of abies and aspen wood [60,61]. When ethanollignin is extracted from xylan-free aspen wood, its polydispersion decreases down to 2.78 compared to that of ethanollignin extracted from initial aspen wood (4.15). Profiles of the molecular mass distribution (Figure 4) demonstrate a higher uniformity of the ethanollignin extracted from xylan-free aspen wood.

There is a minor shoulder in the low molecular region (~500 g/mol) in the curve related to the ethanollignin extracted from the initial aspen wood. This can be accounted for by the extraction of not only the ethanollignin but also the low-molecular fractions of xylan during the wood treatment with ethanol. This is why the clearly defined monomodal shape is seen in the profile of the molecular mass distribution of the ethanollignin from the xylan-free aspen wood.

The results obtained lead us to conclude that the alkali pretreatment of aspen wood aimed at xylan extraction has only a minor impact on the composition and structure of the ethanollignin obtained at the next stage. Unlike technical lignins [13], the organosolvent lignins are free of sulfur, are more reactive, and have a less condensed structure [62]. Therefore, organosolv lignins can be widely used for the production of new materials, in-demand chemicals, and biofuels. In particular, ethanollignin was used for the preparation of enterosorbents [53] and nanoporous carbon materials [63] for the production of monomer phenol and aromatic compounds [36].

### 3.4. The Production, Composition, Structure and Thermochemical Properties of Cellulose Products

The traditional multistage method for the production of microcrystalline cellulose (MCC) includes stages of the wood delignification, the bleaching of the cellulose product, and the hydrolysis of the amorphous constituent of the cellulose with mineral acids. The catalytic peroxide delignification of the wood allows for the one-stage production of MCC under milder conditions (100 °C, atmospheric pressure (99.591 kPa)) [64].

Various methods for the production of MFC and NFC are described in the literature, among which are mechanical (cryodisintegration, high-pressure homogenization, ultrasonic treatment, ball mill, ultrafine milling), chemical (TEMPO oxidation, ionic liquids, supercritical treatment with carbon dioxide, acid hydrolysis) and biological (enzyme hydrolysis) methods [65,66]. In the present study, the MFC and NFC were prepared using an ultrasonic treatment, and sulfuric acid hydrolysis was used to remove residual hemicelluloses and amorphous cellulose in order to reduce the degree of cellulose polymerization.

FTIR, XRD, atomic force microscopy, dynamic light scattering techniques, as well as thermal, elemental and chemical analyses were used for the characterization of the cellulose product, MCC, MFC, and NFC obtained in this work from aspen wood. Table 4 shows the data of the chemical analysis of the cellulose product (CP) formed upon the extraction of xylan and ethanollignin from the aspen wood, and of MCC obtained via the catalytic peroxide delignification of the cellulose product.

The content of hemicelluloses in the wood decreases from 19.2 wt.% to 8.6 wt.%, and the content of lignin decreases from 19.3 wt.% to 8.9 wt.% after the extraction of xylan and ethanollignin. The content of cellulose is 78.4 wt.% in the extracted wood, and 87 wt.% in MCC. A certain amount of hemicelluloses, firmly bound to the cellulose matrix, remains in the MCC, which is typical for the processes of catalytic peroxide delignification of wood in the “acetic acid–water” medium [7].

From the data regarding the elemental analysis (Table 5) the MCC sample was determined to be free of sulfur. The occurrence of sulfur in MFC (2.8 wt.%) and NFC (0.07 wt.%) is due to the formation of sulfo groups during MCC treatment with sulfuric acid.

**Figure 5:**
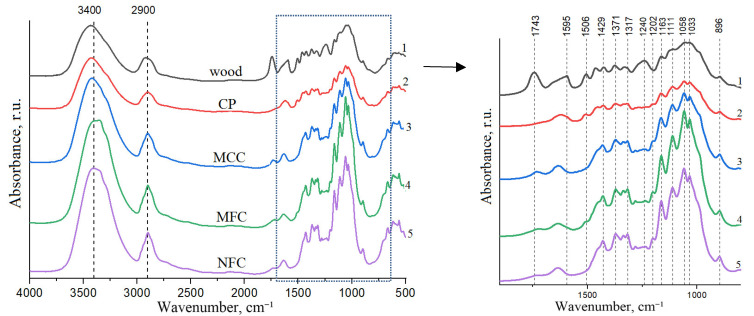
FTIR spectra of the initial aspen wood (1), cellulose product (2), MCC (3), MFC (4), and NFC (5).

The FTIR spectra of the cellulose product separated from the aspen wood is shown in Figure 5. The band of stretching vibrations of OH-groups at 3400 cm^−1^ is narrowed in the spectra of MCC and NFC, most likely due to the removal of amorphous polysaccharides [67]. In the IR spectrum of the cellulose product formed after the extraction of xylan and ethanollignin from the aspen wood, the absorption bands assigned to the stretching vibrations of hemicelluloses (1743 cm^−1^ and 1240 cm^−1^) and lignin (1506 cm^−1^) are less intense. The absorption bands characteristic of lignin and hemicelluloses are not observed in the spectra of MFC and NFC; hence, these products either do not contain no lignin or hemicelluloses or only contain them in negligible quantities. The absorption band at 1429 cm^−1^ related to the bending vibrations of CH_2_ is called the crystallinity band in cellulose [68]. This band is more intense in the spectra of MCC, MFC and NFC than in the spectra of the initial wood and CP. The band at 896 cm^−1^ related to the bending vibrations of glycoside bonds is called the amorphous band. The presence of a.b. at 1202 cm^−1^ that is characteristic of sulfo groups [69] indicates the formation of SO_3_H-groups in cellulose.

**Figure 6:**
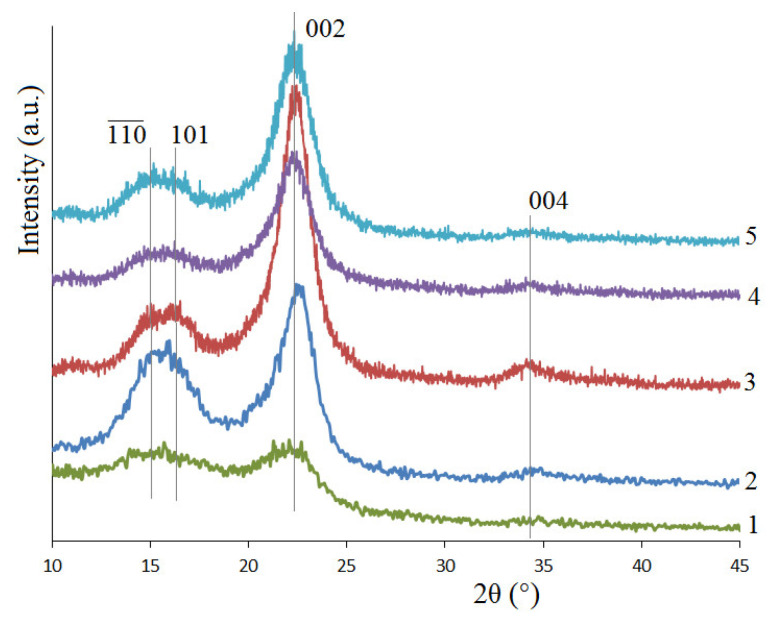
XRD patterns of initial wood (1), cellulose product (2), MCC (3), MFC (4), and NFC (5).

The XRD technique was used for the characterization of the crystal structure of cellulose. The maxima at the angles of 15.0°, 16.4°, 22.3° and 34.2° in XRD patterns of CP, MCC, MFC and NFC (Figure 6) indicate reflections from planes ($\overset{—}{110}$), (101), (002) and (004) of the cellulose crystal lattice I.

XRD studies revealed that the removal of hemicelluloses, lignin, and amorphous cellulose from the wood results in an increase in the crystallinity index in the series: aspen wood < CP < MCC < MFC < NFC (Table 6), while the polymerization degree decreases in the same series (Table 6). The crystallinity indices are identical in the MCC under study, and in the MCC obtained via peroxide delignification of the initial aspen wood [7]. The high crystallinity index of MFC is provided due to the removal of the residual amorphous cellulose during acid hydrolysis and ultrasonic treatment. The crystallinity index of MFC is comparable to that of the cellulose produced by the TEMPO-oxidation of bleached pine kraft pulp (0.84 and 0.87, respectively, depending on the oxidation conditions) [33]. The crystallinity index of NFC from aspen wood (0.86) is close to that of NFC from bark (*Helicteres isora*) (0.9) [70], and NFC from bleached bluegum kraft pulp (0.81–0.83) [71].

**Figure 7:**
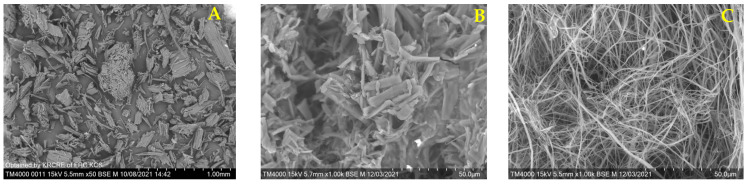
SEM images of MCC (**A**), MFC (**B**), and NFC (**C**) from aspen wood.

Scanning electron microscopy was used for studying the morphology of MCC, MFC, and NFC (Figure 7).

Particles of various MCC are known to vary in size from 1 to 400 µm depending on the nature of the initial cellulose material and on the process conditions [72]. The MFC sample comprises aggregates of 4 to 25 µm in length and 2 to 6 µm in thickness. A spider-like fiber structure is characteristic of NFC that is typical of nanofibrillated cellulose materials. The fiber bundles are 0.4 to 4 µm in diameter and more than dozens of micrometers in length. Nanofibrils supposedly bind to each other to form aggregated bundles during freeze drying. A similar morphology was observed with the nanofibrils obtained from *Stipa Tenacissima* [73].

**Figure 8:**
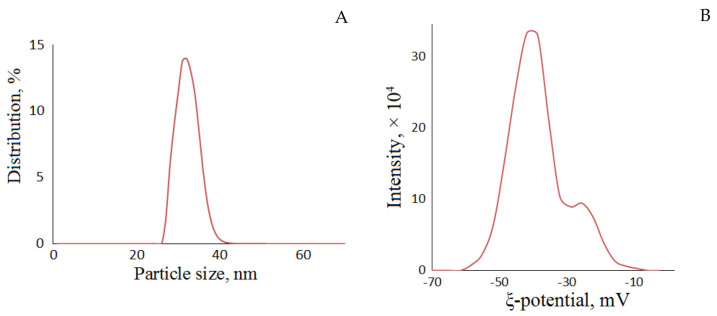
Hydrodynamic diameter of particles (**A**) and the zeta potential (**B**) of the NFC suspension in water.

The size distribution of NFC particles was studied using dynamic light scattering (DLS). In terms of DLS, particles are considered as spherical molecules to determine their average diameter. Even though this is not an accurate analysis for fibrils and rod-like molecules, the method can be used as evidence of the nanometer size of the particles [74]. The NFC particles were established to be predominantly 25 to 42 nm in size, the average hydrodynamic diameter being 36.6 nm (Figure 8A). Approximately the same hydrodynamic diameters (40.8 and 42.4 nm) were observed with cellulose nanofibrils produced from pineapple pomaces [75].

The colloidal stability of NFC in aqueous media is an important property that makes it applicable in aqueous suspension media. The stability depends on the zeta-potential of NFC dispersion in water [76]. The suspension is stable due to the electrostatic repulsion between nanofibrils at a zeta potential below −30 mV [69]. A zeta potential equal to −41.5 mV was established for the suspension of NFC from aspen wood that is comparable to the data found in the literature [77] on the zeta potential of TEMPO-oxidized nanofibers produced from bleached softwood kraft pulp (−42 mV).

This means that the NFC under study has a negatively charged surface as a result of the formation of sulfo groups during MCC treatment with sulfuric acid. From the elemental analysis data, the NFC sample was determined to contain 0.07% sulfur.

### 3.5. Structure of the NFC Film

Nanocellulose films are promising for applications in medicine [78], packaging materials [79], and other areas [80].

**Figure 9:**
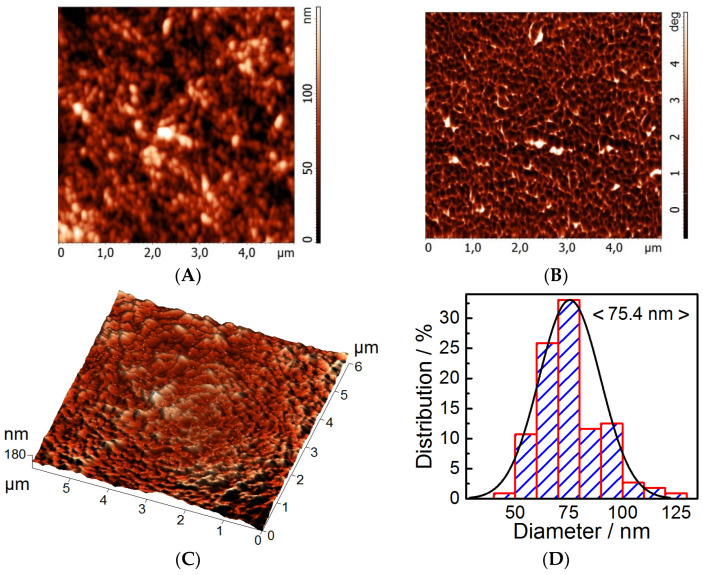
AFM images of NFC film from aspen wood: relief (**A**), phase contrast (**B**), 3D relief (**C**), and particle size distribution (**D**).

Atomic force microscopy was used for studying the structure of NFC films produced from aspen wood. The film relief is seen (Figure 9A) to be composed of rather uniform particles of 75.4 nm in average diameter. The phase contrast image indicates no foreign inclusions in the film surface (Figure 9B). The white regions in the phase contrast image belong to the graphite support. The grained morphology and larger particle diameters (against the hydrodynamic diameter in the solution) are thought to result from the fibril intertwining during the suspension drying in order to obtain the film.

The characteristics of the NFC film surface agree with the data from the literature [70,80]. The height difference on the film surface equal to 180 nm is similar to that observed with the NFC film obtained from *Helicteres isora* [70]. The average roughness is 41.5 nm in the NFC film from aspen cellulose, but 76.6 nm was produced in the NFC film upon the substitution of dimethyladipate for water in the NFC suspension [80].

### 3.6. Thermal Properties of MCC, MFC, and NFC

**Figure 10:**
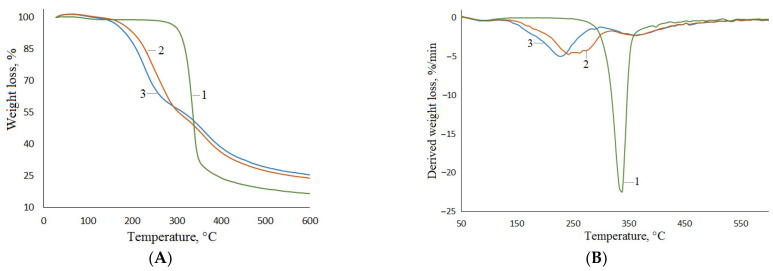
TG (**A**) and DTG (**B**) curves of MCC (1), MFC (2), and NFC (3).

The thermal properties of nanocellulose materials are important when they are used as reinforcing components in composite materials. A thermogravimetric analysis was used for studying the thermal transformations of MCC, MFC and NFC produced from aspen wood. Thermogravimetric curves (Figure 10) show that minor weight loss is observed with all of the samples at temperatures as low as 100 °C due to the release of the sorbed water. The main process of the thermal degradation of MCC starts above 280 °C; it proceeds in one stage which corresponds to the peak at 336 °C in the DTG curve. The processes of cellulose dehydration and depolymerization, as well as the destruction of residual hemicelluloses, occur at this temperature [68].

The intensive two-stage thermodestruction of NFC and MFC starts at lower temperatures (120 and 150 °C, respectively) (Figure 10A). These stages are indicated by two broad peaks in the DTG curves (Figure 10B), with maxima at 227 and 361 °C in the case of NFC and at 242 and 362 °C in the case of MFC. The first stage is the cleavage of glycoside bonds accompanied by cellulose dehydration. At the second stage (above 340 °C), reactions of depolymerization begin to dominate; upon further heating, these processes cause the amorphization of the cellulose structure to be transformed to the carbonized structure of carbon [81]. A similar process was described for the thermal decomposition of nanofibrils obtained from pine kraft pulp: two thermodestruction regions were observed at 225 and 380 °C [82]. Against the decomposition of MCC, the intensive decomposition of NFC and MFC starts at a lower temperature that is accounted for by smaller particles and the presence of sulfo groups which behave as dehydration catalysts [83].

The obtained thermogravimetric results were used for the calculation of the activation energy of the main period of the thermal decomposition of MCC, MFC and NFC. In view of the fact that the pyrolysis of cellulose includes a number of simultaneous and/or consecutive chemical reactions accompanied by weight loss, the principal kinetic characteristic of the pyrolysis process, the activation energy, should be treated as an effective or apparent parameter.

**Figure 11:**
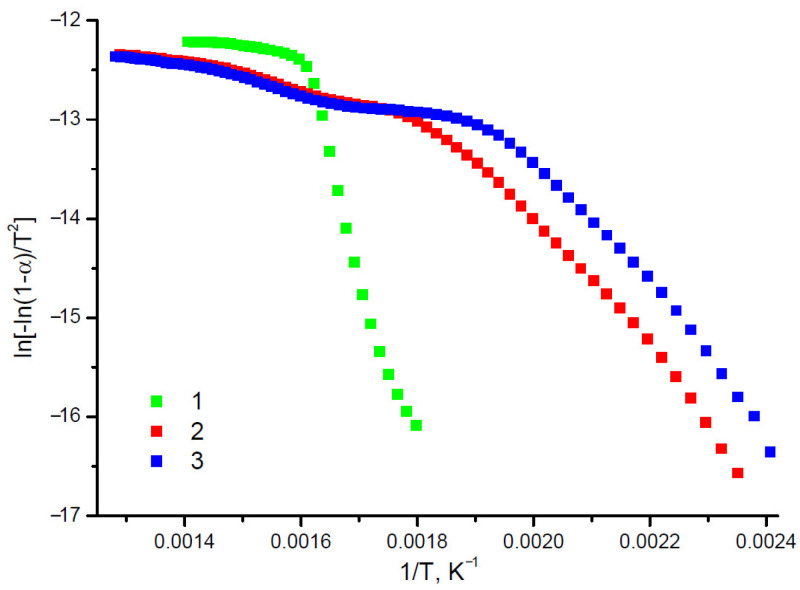
Coats-Redfern linearization of experimental thermogravimetric data: MCC (1), MFC (2), and NFC (3).

The Coats-Redfern [45,46] linearization of the thermogravimetric data on the cellulose samples is illustrated in Figure 11.

The shape of the kinetic curves argues for two mechanisms of the main decomposition of the cellulose samples under study. The sharp rise of the curves is thought to characterize the cellulose destruction. The plateau indicates the amorphization of its structure and the formation of a coke residue. The inflection point in the curves is a conditional boundary between these processes. The shapes of the kinetic curves of MFC and NFC are, in general, similar to one another, but different from the curve of MCC. The temperature ranges of the main stages of decomposition and the apparent activation energies are summarized in Table 7.

The tabulated data show a considerable widening of the temperature range of the main decomposition of post-hydrolysis samples (152–302 and 117–242 °C for MFC and NFC, respectively). A probable reason is the presence of several structural components decomposed at similar temperatures.

The main decomposition of MCC is observed in a temperature range (283–438 °C), and needs a considerable activation energy (166 kJ/mol). This is accounted for by the regular linear structure of cellulose macromolecules, the crystal structure, and strong hydrogen bonds. As temperature rises, condensation processes that do not require high energy consumption start to dominate.

The destruction of MFC and NFC consumes far less energy (50 and 64 kJ/mol, respectively). This is due to the presence of sulfo groups in the MFC and NFC structures. It is known [82,83] that sulfate groups catalyze dehydration and, as a consequence, decrease the activation energy and facilitate the thermal decomposition of the nanocellulose material.

The results of the performed study make a new contribution to the solution of an urgent and important problem: the development of effective and environmentally friendly methods of fractionation of a lignocellulose biomass into the required range of target chemical products. One of the ways to solve this problem is by the integration of various catalytic processes that ensure the processing of all of the main components of the biomass.

In particular, we proposed to carry out the fractionation of birch wood into MCC, MFC, NFC, xylose, and adsorbents using heterogeneous catalytic processes of the hydrolysis of hemicelluloses of wood to xylose and the peroxide delignification of “hemicelluloses-free” wood into MCC [84]. Methods combining extraction and catalytic processes make it possible to produce a different set of chemical products from wood. The integration of the processes of the extraction isolation of xylan and ethanol lignin from birch wood and the processes of catalytic hydrolysis of xylan and cellulose makes it possible to produce xylose from xylan, levulinic acid from cellulose, and enterosorbents from ethanol lignin [53].

In this work, an extraction-catalytic method of fractionation of aspen wood biomass was developed which allows for the obtaining of MCC, MFC, NFC, and the xylan and ethanol lignin biopolymers. These chemical products are in demand in many areas.

## 4. Conclusions

A new method of extraction, the catalytic fractionation of aspen wood into microcrystalline, microfibrillated, nanofibrillated celluloses, and xylan and ethanol lignin, was developed. This method ensures the utilization of all main components of the woody biomass by integrating the processes of alkaline and ethanol extraction, catalytic peroxide delignification, sulfuric acid hydrolysis, and ultrasonic treatment.

Peroxide delignification in the “acetic acid-water-sulfuric acid catalyst” medium of the lignocellulose product that is formed after the extraction isolation of xylan and ethanol lignin from aspen wood allows for the obtaining of MCC that contains a very small amount of residual lignin.

The sulfuric acid hydrolysis of MCC, followed by ultrasonic treatment and centrifugation, is used for the production of MFC and NFC. The yields of the products under optimized conditions of the extractive-catalytic fractionation of aspen wood were (wt.%): 10.2 for xylan, 42.8 for MCC, 14.4 for MFC, 19.0 for NFC, and 11.2 for ethanollignin.

The compositions and structures of the products obtained from aspen wood were characterized using elemental and chemical analysis, FTIR, XRD, GC, GPC, SEM, AFM, DLS, and DTA methods.

The products obtained are in demand in many application areas. Xylan is used for the production of xylose, furfural, polyatomic alcohols and organic acids, as well as for the creation of new composite materials, including bioplastics and coatings. Ethanollignin can be used for the production of sorbents, phenol compounds, and liquid biofuels. MCC is widely used in the pharmaceutical and food industries. MFC and NFC have broad prospects for use in the production of composite materials for various purposes, in electronics, and in medicine and other fields.

## Figures and Tables

**Table 1:** Elemental analysis of xylan obtained in this study and the results reported in the literature.

Sample	Elemental Composition, wt.%			Atomic Ratio H/C	Atomic Ratio O/C
	C	H	O_dif_		
Xylan from aspen wood	39.13 ± 0.04	5.89 ± 0.03	54.97 ± 0.07	1.81	1.05
Xylan from birch wood [53]	39.14	5.88	54.98	1.80	1.05
Commercial xylan [52]	40.68	6.68	52.65	1.98	0.97

**Table 2:** The elemental composition of ethanollignins from aspen and poplar wood.

Sample *	Elemental Composition, wt.%			Atomic Ratio H/C	Atomic Ratio O/C
	C	H	O_dif_		
1	63.67 ± 0.5	5.95 ± 0.04	30.37 ± 0.54	1.1	0.4
2	62.1	5.6	31.8	1.1	0.4

* 1: extracted from xylan-free aspen wood; 2: extracted from poplar wood [58].

**Table 3:** The molecular mass distribution of ethanollignin from aspen wood.

Sample	Mn (g/mol)	Mw (g/mol)	PD
1	972 ± 2	2704 ± 6	2.78 ± 0.01
2	659 ± 3	2738 ± 7	4.15 ± 0.01

1: extracted from xylan-free wood; 2: extracted from initial aspen wood.

**Table 4:** Chemical composition of the cellulose product and the MCC produced from aspen wood.

Sample	Component Content, wt.%			
	Cellulose	Lignin	Easy Hydrolysable Polysaccharides	Ash
CP	78.4 ± 0.8	8.9 ± 0.1	8.6 ± 0.2	0.7 ± 0.01
MCC	87.0 ± 0.8	0.4 ± 0.005	8.5 ± 0.2	0.8 ± 0.01

**Table 5:** Elemental composition of MCC, MFC and NFC obtained from aspen wood.

Sample	Elemental Composition, wt.%				Atomic Ratio H/C	Atomic Ratio O/C
	C	H	S	O_dif_		
MCC	42.6 ± 0.04	6.1 ± 0.01	-	51.2 ± 0.06	1.7	0.9
MFC	42.3 ± 0.01	6.1 ± 0.01	2.8 ± 0.09	48.6 ± 0.07	1.8	0.9
NFC	41.8 ± 0.06	6.1 ± 0.02	0.07 ± 0.005	51.9 ± 0.08	1.8	0.9

**Table 6:** Crystallinity index (CrI) and polymerization degree of cellulose product samples obtained from aspen wood.

Sample	CrI	Polymerization Degree
CP	0.65	405
MCC	0.73	313
MFC	0.86	176
NFC	0.86	89

**Table 7:** Activation energy of the thermal decomposition of cellulose samples at the stage of main destruction.

Sample	Temperature Range, °C	Activation Energy, kJ/mol	Correlation Coefficient, R2
MCC	283–358	166	0.98
	363–438	6	0.97
MFC	152–302	50	0.98
	307–502	11	0.97
NFC	117–242	64	0.97
	247–507	10	0.98

## Data Availability

Not applicable.
